# Differential Stimulation of Insulin Secretion by GLP-1 and Kisspeptin-10

**DOI:** 10.1371/journal.pone.0113020

**Published:** 2014-11-17

**Authors:** Tara A. Schwetz, Christopher A. Reissaus, David W. Piston

**Affiliations:** 1 Department of Molecular Physiology and Biophysics, Vanderbilt University, Nashville, Tennessee, United States of America; 2 Department of Physics, Vanderbilt University, Nashville, Tennessee, United States of America; 3 Department of Chemical and Biomolecular Engineering, Vanderbilt University, Nashville, Tennessee, United States of America; Johns Hopkins University School of Medicine, United States of America

## Abstract

β-cells in the pancreatic islet respond to elevated plasma glucose by secreting insulin to maintain glucose homeostasis. In addition to glucose stimulation, insulin secretion is modulated by numerous G-protein coupled receptors (GPCRs). The GPCR ligands Kisspeptin-10 (KP) and glucagon-like peptide-1 (GLP-1) potentiate insulin secretion through G_q_ and G_s_-coupled receptors, respectively. Despite many studies, the signaling mechanisms by which KP and GLP-1 potentiate insulin release are not thoroughly understood. We investigated the downstream signaling pathways of these ligands and their affects on cellular redox potential, intracellular calcium activity ([Ca^2+^]_i_), and insulin secretion from β-cells within intact murine islets. In contrast to previous studies performed on single β-cells, neither KP nor GLP-1 affect [Ca^2+^]_i_ upon stimulation with glucose. KP significantly increases the cellular redox potential, while no effect is observed with GLP-1, suggesting that KP and GLP-1 potentiate insulin secretion through different mechanisms. Co-treatment with KP and the G_βγ_-subunit inhibitor gallein inhibits insulin secretion similar to that observed with gallein alone, while co-treatment with gallein and GLP-1 does not differ from GLP-1 alone. In contrast, co-treatment with the G_βγ_ activator mSIRK and either KP or GLP-1 stimulates insulin release similar to mSIRK alone. Neither gallein nor mSIRK alter [Ca^2+^]_i_ activity in the presence of KP or GLP-1. These data suggest that KP likely alters insulin secretion through a G_βγ_-dependent process that stimulates glucose metabolism without altering Ca^2+^ activity, while GLP-1 does so, at least partly, through a G_α_-dependent pathway that is independent of both metabolism and Ca^2+^.

## Introduction

Insulin secretion is tightly regulated to maintain glucose homeostasis. During glucose stimulated insulin secretion (GSIS) from pancreatic β-cells, glucose is metabolized to increase the ATP/ADP ratio, which inhibits the ATP-sensitive inward rectifying potassium (K_ATP_) channel. The β-cell is subsequently depolarized, which activates voltage-gated calcium channels (VGCC) and stimulates insulin secretion [1]. Beyond GSIS, multiple G-protein coupled receptor (GPCR) ligands also play a large role in the modulation of insulin release [2]. Since GPCRs are common therapeutic targets and constitute about 50% of drugs on the market [3], a thorough understanding of the mechanisms by which GPCR ligands modulate insulin release is crucial.

Originally identified as a metastasis suppressor gene in breast cancer and melanoma cell lines, the KISS1 gene products – kisspeptins – have been identified as the endogenous ligands for GPR-54, expression of which has been detected in pancreatic islets. Specifically, mRNA expression of kisspeptin (KP) and GPR-54 has been observed in mouse and human islets and both co-localize with murine insulin and glucagon positive cells [4], [5]. Activation of GPR-54, a G_q_-coupled receptor that stimulates the phospholipase-c (PLC) pathway, has been shown to potentiate insulin release from human and mouse islets [6], [7] although this effect remains debated [8], [9].

GLP-1 is a potent stimulator of insulin secretion. GLP-1 is an incretin hormone secreted by the L-cells of the distal intestine, and it binds to the G_s_ coupled GLP-1 receptor, GLP-1R [10]. GLP-1 has been shown to induce effects on multiple organ systems, including the heart, brain, and liver [11]. In the pancreas, GLP-1 stimulates insulin gene expression and proinsulin biosynthesis, in addition to its potentiation of GSIS. GLP-1 also has proliferative and anti-apoptotic effects on the β-cell [12], [13]. Patients with type 2 diabetes mellitus (T2DM) display impaired GLP-1 secretion and/or responses. Because of GLP-1′s modulation of pancreatic hormones (increased insulin and decreased glucagon release), it has developed into a viable candidate for the treatment of T2DM. GLP-1R agonists have been utilized to efficiently decrease hemoglobin (Hb)A_1c_ levels in patients with T2DM [14].

The general mechanisms by which KP and GLP-1 potentiate insulin have been determined; however, the detailed pathways activated by these ligands in pancreatic β-cells, particularly by KP, remain relatively unclear. Here, we investigated the effect of KP and GLP-1 on murine cellular redox potential, Ca^2+^ signaling, and insulin secretion to determine the downstream pathways by which these ligands function.

## Materials and Methods

### Islet Isolation

All murine procedures were approved by and conducted in compliance with the Vanderbilt University Institutional Animal Care and Use Committee (IACUC) operating under Public Health Service Animal Welfare Assurance #A3227-01. All surgery procedures were performed following intraperitoneal injection of ketamine/xylazine (∼60 mg/kg/∼12 mg/kg) anesthesia. Islet isolation protocol was performed as previously described [15]–[17]. Briefly, pancreata from random healthy 8–12 week old C57BL/6 adult male mice (Harlan) were excised and digested in 0.15–0.22% Collagenase P (Roche) per ml of Hank’s Balanced Salt Solution (HBSS; Invitrogen) for 8–12 minutes under gentle agitation. Samples were centrifuged 3x and the supernatant was replaced with fresh HBSS. Individual islets were pipetted into fresh islet media [RPMI 1640 media (Invitrogen) supplemented with 10% fetal bovine serum (FBS; Invitrogen), penicillin/streptomycin (Invitrogen), and 11 mM glucose] and incubated overnight prior to use at 37°C and 5% CO_2_ for 24–48 hours.

### Islet Dispersion

Islets were isolated as described above and allowed to recover overnight. Islets were washed in DPBS (Corning) and dispersed using Accutase (Life Technologies) for 10 minutes at 37°C under gentle agitation. Cells were centrifuged, washed with fresh islet media, and plated on glass bottom micro-well dishes (MatTek). Plated cells were incubated prior to use at 37°C and 5% CO_2_ for 24–48 hours.

### Microfluidic Device Construction

Microfluidic devices were constructed using Sylgard 184 Silicone Elastomer base and curing agent mix (Dow Corning) that was degassed and cured on a master mold for 3 hours at 75°C. A well and two access holes for loading and removing the islets were created. The molds were bonded onto 22×40 mm cover glass (Corning) following plasma cleaning (Harrick Scientific).

### NAD(P)H Imaging

All imaging experiments were conducted in a microfluidic device at 37°C and 5% CO_2_. The imaging buffer consisted of (in mM) 125 NaCl, 5.7 KCl, 2.5 CaCl_2_•2 H_2_O, 1.5 MgCl_2_, 10 HEPES, and 0.1% bovine serum albumin (BSA; Sigma Aldrich) at pH 7.4. NAD(P)H autofluorescence was imaged with a LSM710 microscope (Carl Zeiss) using a Plan-Apochromat 20x/0.8 NA objective and a Coherent Chameleon laser tuned to 710 nm, as previously described [17]. The laser power at the sample was below 3.5 mW to prevent damage to the islet [18]. NAD(P)H autofluorescence was measured in intact islets as a function of glucose concentration (2–23 mM) with and without Kisspeptin-10 (KP; 1 µM; Tocris, Cat. #2570) and GLP-1 7–36 amide (20 nM; Bachem, H-6795). Addition of increasing glucose concentrations with and without the GPCR ligands occurred at 8 min intervals to allow NAD(P)H levels to plateau, after which Z-stacks were collected.

### Imaging of Ca^2+^ Oscillations

Ca^2+^ oscillation experiments were conducted in a microfluidic device (whole islets) or glass bottom micro-well dishes (dispersed β-cells) at 37°C and 5% CO_2_. Intracellular calcium concentration ([Ca^2+^]_i_) oscillation frequency was measured pre- and post-treatment with KP, GLP-1, gallein, or mSIRK individually and in combination, as previously described [17]. Intact islets were labeled with Fluo4-AM (4 µM; Invitrogen) in imaging buffer with 2 mM glucose for 30–45 minutes prior to data collection. Oscillations in Fluo4 fluorescence over the whole islet area were detected by excitation at 488 nm on a LSM 5Live microscope (Carl Zeiss) with a Plan-Apochromat 20x/0.8 NA lens. Images were collected at 1 frame every 2 seconds to measure the fast oscillations in [Ca^2+^]_i_ generated by changes in ion channel conductances (∼25 sec). Cells were imaged at 10 mM glucose for ∼5–10 min to allow sufficient time for synchronous oscillations to appear; the GPCR ligand and/or G_βγ_ modulator of interest then was added and oscillations were continuously recorded for another ∼10 min. Data were normalized to the untreated control frequency or amplitude for each islet prior to addition of a GPCR ligand and/or G_βγ_ modulator.

### Static Incubation Insulin Secretion Assays

Islets were isolated as described above and allowed to recover overnight. Islets were pre-incubated for 1 hour in KRBH buffer consisting of (in mM) 128.8 NaCl, 4.8 KCl, 1.2 KH_2_PO_4_, 1.2 MgSO_4_•7 H_2_O, 2.5 CaCl_2_, 20 Hepes, 5 NaHCO_3_ (pH 7.4) with 2.8 mM glucose. 4 islets per sample were incubated in 1 mL KRBH buffer at 2.8 mM, 10 mM, or 16.7 mM glucose with and without KP, GLP-1, gallein, or mSIRK individually or in combination for 45 minutes at 37°C; each condition was measured in triplicate. The samples were briefly spun at 3000 rpm (Beckman) and 500 µL of each sample were placed in a new tube. 500 µL of 2% Triton-X were added to each islet sample to lyse the islets prior to storage at −20°C. Insulin content (from the Triton-X-containing samples) and secretion were analyzed in duplicate with a Mouse Ultrasensitive Insulin ELISA kit (Alpco) and detected on a Spectra Max M5 spectrometer (Molecular Devices).

### Analysis and Statistics

Data were analyzed with Microsoft Excel, ImageJ, MatLab, or GraphPad Prism software. For all imaging data, the background signal was subtracted and the mean ± S.E. was determined. Student’s *t*-tests and ANOVAs were used where applicable and Welch’s correction was used in cases where variance was statistically different between groups. *p*<0.05 was considered statistically significant unless otherwise noted.

## Results

### Kisspeptin and GLP-1 potentiate insulin secretion at elevated glucose levels

**Figure 1:**
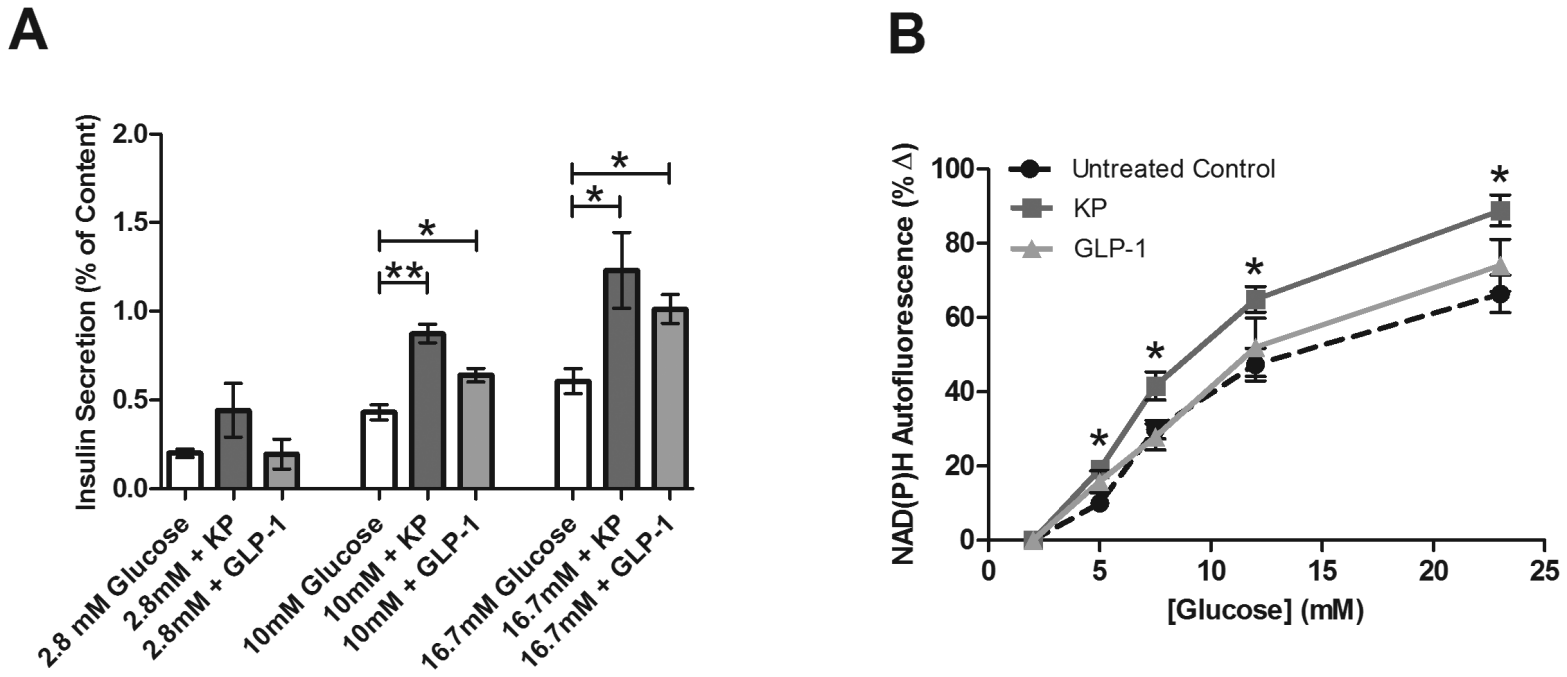
**A**. Percent of insulin content secreted from intact islets after static incubation at 2.8, 10, or 16.7 mM glucose with and without KP (1 µM, dark gray) or GLP-1 (20 nM, light gray). Secretion from untreated control islets is shown in white. Data are the mean ± S.E. *n* = 4–19. *(*p*<0.05) and **(*p*<0.001) indicate significance compared to untreated control. **B**, Glucose-dependent percent change in NAD(P)H from untreated intact islets (circles) and islets treated with KP (1 µM, squares) or GLP-1 (20 nM, triangles) compared to values at 2 mM glucose. Data are the mean ± S.E. *n* = 9–11. *p<0.01.

We first investigated the effect of kisspeptin-10 (KP) and GLP-1 on insulin secretion from intact islets. Although KP is thought to play a role in modulation of insulin secretion, conflicting data have been published as to its effect on β-cell exocytosis [6]–[9], [19]. Here, intact islets were statically incubated with KP (1 µM) or GLP-1 (20 nM) at 2.8 mM, 10 mM, and 16.7 mM glucose concentrations to validate previous results and confirm that KP and GLP-1 stimulate insulin secretion in our system. At sub-stimulatory glucose concentrations (2.8 mM), KP and GLP-1 do not increase insulin secretion compared to the untreated control (0.44±0.15% and 0.20±0.08% vs. 0.19±0.04%, respectively; *p*>0.1), as shown in Figure 1A. At 10 mM glucose, KP and GLP-1 potentiate insulin release compared to untreated control (0.87±0.05% and 0.64±0.03% vs. 0.44±0.04%; *p*<0.001 and p<0.05 respectively). Insulin secretion after incubation at very high, stimulatory glucose levels (16.7 mM) was 0.68±0.11% in the untreated control and this was significantly increased by treatment with KP or GLP-1 (1.23±0.21% and 1.10±0.09%, respectively; *p*<0.03, Fig. 1A), consistent with prior studies [6], [7], [10].

### Kisspeptin increases cellular redox potential, but GLP-1 does not

We next evaluated the effects on cellular metabolism during KP or GLP-1 exposure. While GLP-1′s metabolic effects have been examined [20], little has been reported about the effect of KP on cellular metabolism, except that kisspeptin gene products may play an important role in sensing body energy status [21]. To determine whether KP or GLP-1 effects on insulin secretion are correlated with changes in the metabolic processing of glucose, we used NAD(P)H autofluorescence as a measure of cellular redox potential [22], [23]. NAD(P)H levels from intact islets were measured as a function of glucose in the presence and absence of KP or GLP-1. In untreated control islets, increasing concentrations of glucose produce a rise in the NAD(P)H autofluorescence and cellular redox potential (Fig. 1B); this is consistent with previously published data from our lab [22]. Treatment with KP, which stimulates insulin secretion through activation of the PLC pathway, resulted in a significant, additional increase in NAD(P)H compared to untreated control islets over a range of glucose concentrations (*p*<0.01, Fig. 1B). However, treatment with GLP-1 did not significantly increase NAD(P)H autofluorescence compared to untreated control islets. These GLP-1 data are consistent with a previously published study [20].

### Kisspeptin does not affect calcium signaling in intact and dispersed islets

Insulin secretion and Ca^2+^ signaling are tightly coupled processes. Islet β-cells display synchronous oscillations in their intracellular Ca^2+^ activity ([Ca^2+^]_i_) at glucose concentrations greater than 7 mM, resulting in pulsatile release of insulin [24], [25]. We used a calcium indicator dye, Fluo4, to measure the β-cell Ca^2+^ response in isolated islets upon treatment with KP.

**Figure 2:**
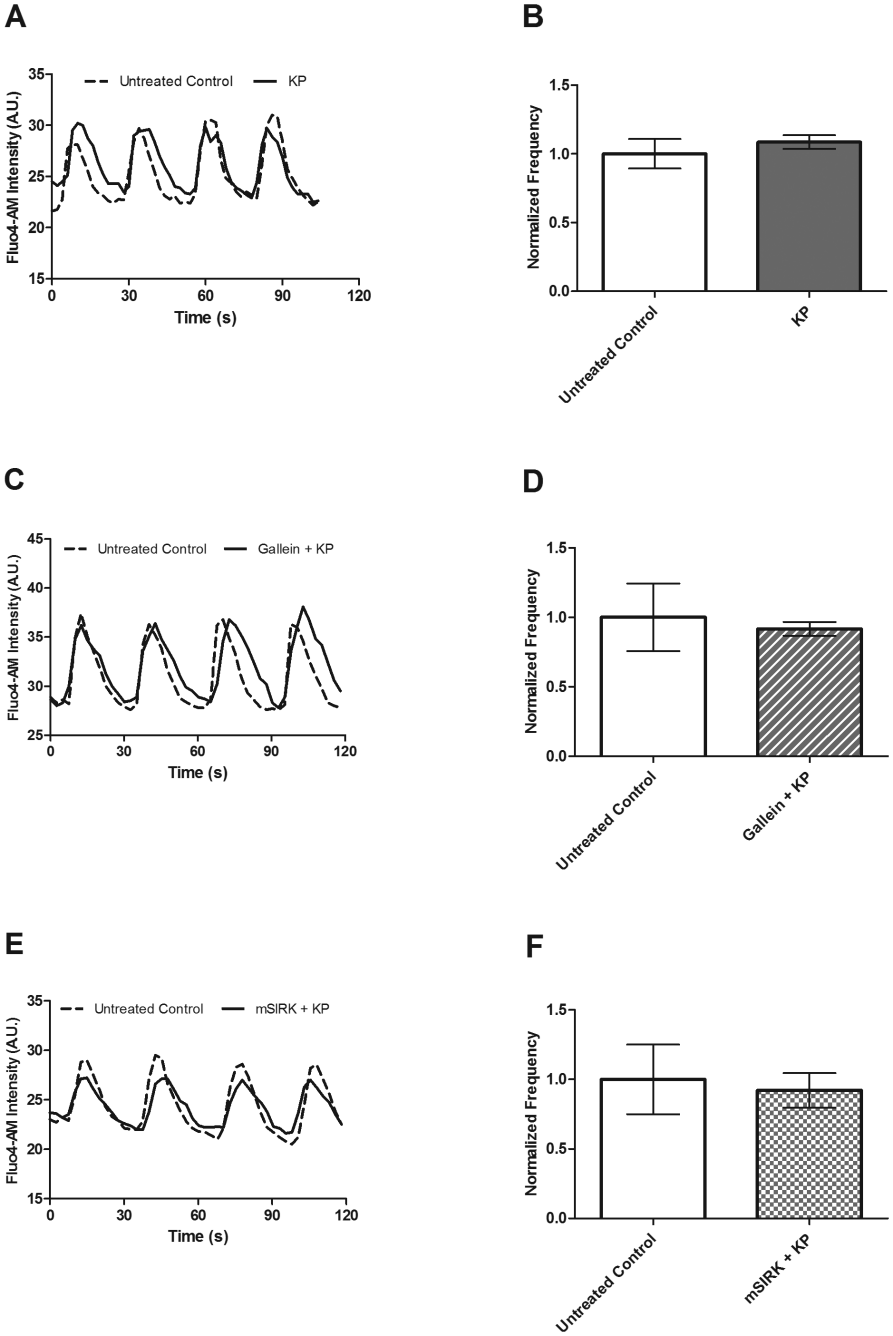
**A**. Changes in Fluo4 signal recorded at 10 mM glucose in the presence and absence of KP (1 µM) alone or in combination with gallein (10 µM) or mSIRK (30 µM) to measure the frequency and amplitude of [Ca^2+^]_i_ oscillations. **A, C, & E**, Representative oscillations in [Ca^2+^]_i_ recorded from intact islets before (dotted line) and after (solid line) treatment with KP (**A**), gallein+KP (**C**), or mSIRK+KP (**E**). **B, D & F**, The normalized frequency of [Ca^2+^]_i_ oscillations pre- and post-treatment with KP (**B**), gallein+KP (**D**), or mSIRK+KP (**F**). Data following addition of KP (dark gray), gallein+KP (dark gray stripes), or mSIRK+KP (dark gray checks) are normalized to the data collected from the islet prior to treatment. Data are the mean ± S.E. *n* = 4–5. *p*>0.4.

**Figure 3:**
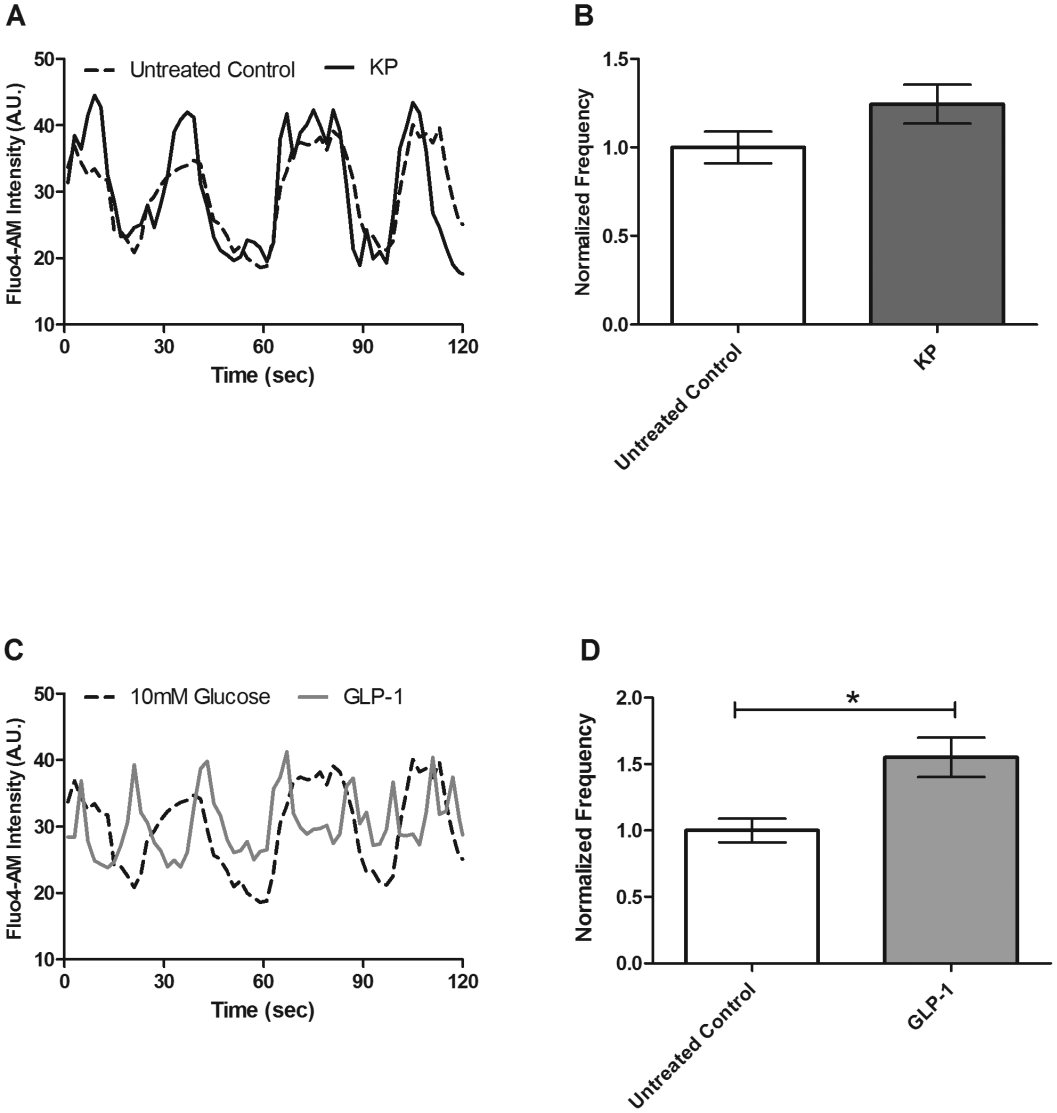
Changes in Fluo4 signal in dispersed β-cells recorded at 10 mM glucose in the presence and absence of KP (1 µM) or with GLP-1 (20 nM) to measure the frequency and amplitude of [Ca^2+^]_i_ oscillations. **A & C**, Representative oscillations in [Ca^2+^]_i_ recorded from dispersed β-cells before (dotted line) and after (solid line) treatment with KP (**A**) or GLP-1 (**C**). **B & D**, The normalized [Ca^2+^]_i_ oscillation frequency measured pre- and post-treatment with KP (**B**, dark gray) or GLP-1 (**D**, light gray). Data are normalized to the data collected from the dispersed β-cells prior to ligand treatment (white). Data are the mean ± S.E. *n* = 4. *p*<0.05.

At 10 mM glucose, an increase in the calcium signal is initially detected, followed by oscillations in [Ca^2+^]_i_, in intact islets. No oscillations were detected at low (2 mM) glucose concentrations. We measured intact islet [Ca^2+^]_i_ oscillations at 10 mM glucose and found no significant effect of KP on either the frequency (39.37±4.21 vs. 42.23±4.11 mHz, *p*>0.6, Fig. 2A & B) or amplitude oscillating component (data not shown). In order to distinguish the effects of these GPCR ligands on [Ca^2+^]_i_ activity, these experiments also were performed in dispersed β-cells for ease of comparability between our study and others [7]. In dispersed β-cells, [Ca^2+^]_i_ oscillations were measured at 10 mM glucose in the presence of KP and no significant effect of KP on either frequency (39.50±3.55 vs. 44.22±4.35 mHz, *p*>0.1, n = 4, Fig. 3A & B) or the amplitude oscillating component was found (data not shown).

As previously described, the KP receptor, GPR-54, is a GPCR. Upon ligand binding to GPCRs, intracellular G-proteins are activated and signal through the G_α_ subunit or the G_βγ_ complex [26]–[28]. Thus, [Ca^2+^]_i_ oscillations following combination treatment with KP and two G_βγ_ modulators were measured. Gallein is a cell-permeable blocker of G_βγ_-dependent activities, while mSIRK, an N-myristoylated G_βγ_-binding peptide, binds to and dissociates G_βγ_ without G_α_ activation. Previously, our lab showed that gallein alone does not significantly alter the Ca^2+^ response [17]; however, mSIRK significantly enhances it, consistent with a prior report [29]. Similar to KP alone, combination treatment with KP and gallein (41.15±9.99 vs. 36.78±7.34 mHz) or mSIRK (39.79±9.97 vs. 34.94±8.98 mHz) does not significantly modulate the frequency (Fig. 2C–F) or amplitude (data not shown) of [Ca^2+^]_i_ oscillations.

### The Ca^2+^ response is not altered upon GLP-1 treatment in whole islets

**Figure 4:**
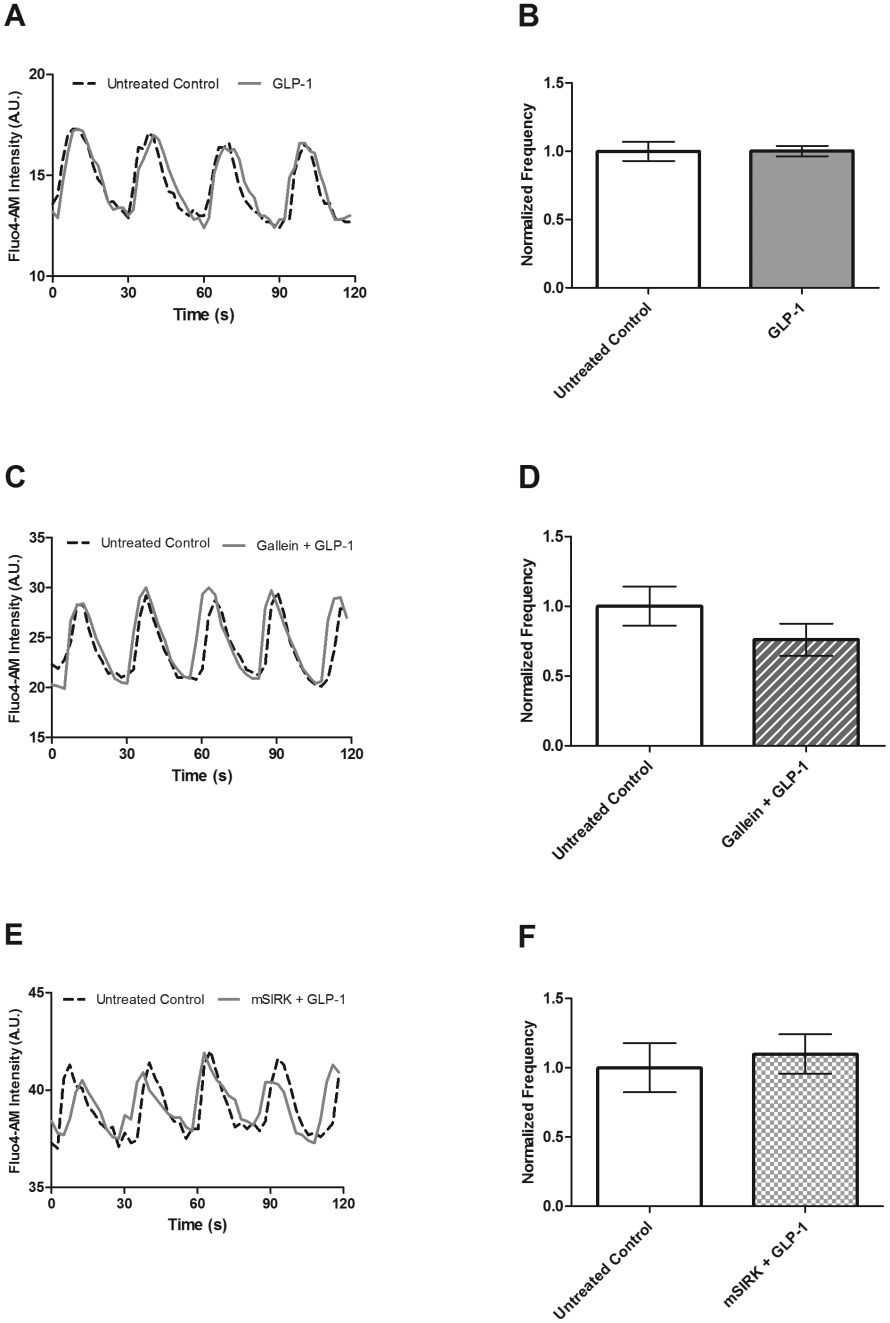
Fluo4 signal recorded at 10 mM glucose in the presence and absence of GLP-1 (20 nM) alone or combined with gallein (10 µM) or mSIRK (30 µM) to detect changes in the frequency and amplitude of [Ca^2+^]_i_ oscillations. **A, C &, E**, Representative [Ca^2+^]_i_ oscillations from intact islets recorded pre- (dotted line) and post-treatment (solid line) with GLP-1 (**A**), gallein and GLP-1 (**C**) or mSIRK and GLP-1 (**E**). **B, D, & F**, The normalized [Ca^2+^]_i_ oscillation frequency measured pre- and post-treatment with GLP-1 (**B**, light gray), gallein and GLP-1 (**D**, light gray stripes) or mSIRK and GLP-1 (**F**, light gray checks). Data are normalized to the data collected from the islet prior to ligand/G_βγ_ modulator treatment (white). Data are the mean ± S.E. *n* = 4–5. *p*>0.1.

GLP-1 previously has been shown to stimulate the Ca^2+^ response from isolated and tissue culture β-cells [30], [31]. To further test this effect in intact islets, the frequency and amplitude of [Ca^2+^]_i_ oscillations were measured pre- and post-treatment with GLP-1 at 10 mM glucose. Similar to the results observed with KP, we did not find GLP-1 to significantly alter the [Ca^2+^]_i_ oscillation frequency (38.91±2.71 vs. 37.94±1.95 mHz, *p*>0.2, Fig. 4A & B) or amplitude (data not shown).

The GLP-1 receptor, GLP-1R, is G_s_-coupled [10]. The effects of combination treatment with GLP-1 and gallein or mSIRK on the Ca^2+^ response were determined. Gallein and GLP-1 co-treatment did not significantly alter either the frequency (47.50±6.68 vs. 35.08±6.07 mHz, *p*>0.7, Fig. 4C & D) or the amplitude of the oscillating component (data not shown). Similarly, combined treatment with mSIRK and GLP-1 also did not alter [Ca^2+^]_i_ oscillations (47.72±8.42 vs. 50.66±7.88 mHz, *p*>0.7, Fig. 4E & F).

### GLP-1 treatment alters the Ca^2+^ response in dispersed beta cells

GLP-1 previously has been shown to stimulate the Ca^2+^ response from dispersed and tissue culture β-cells [32]–[33]. To further test this effect in dispersed islets, the frequency and amplitude of [Ca^2+^]_i_ oscillations were measured pre- and post-treatment with GLP-1 at 10 mM glucose. Similar to previous publications, we found that GLP-1 significantly alters the [Ca^2+^]_i_ oscillation frequency (39.50±3.55 vs. 61.30±5.92 mHz, *p*<0.05, n = 4, Fig. 3C & D), but does not alter the amplitude component (data not shown).

### Kisspeptin stimulates insulin secretion through a G_βγ_-dependent pathway

**Figure 5:**
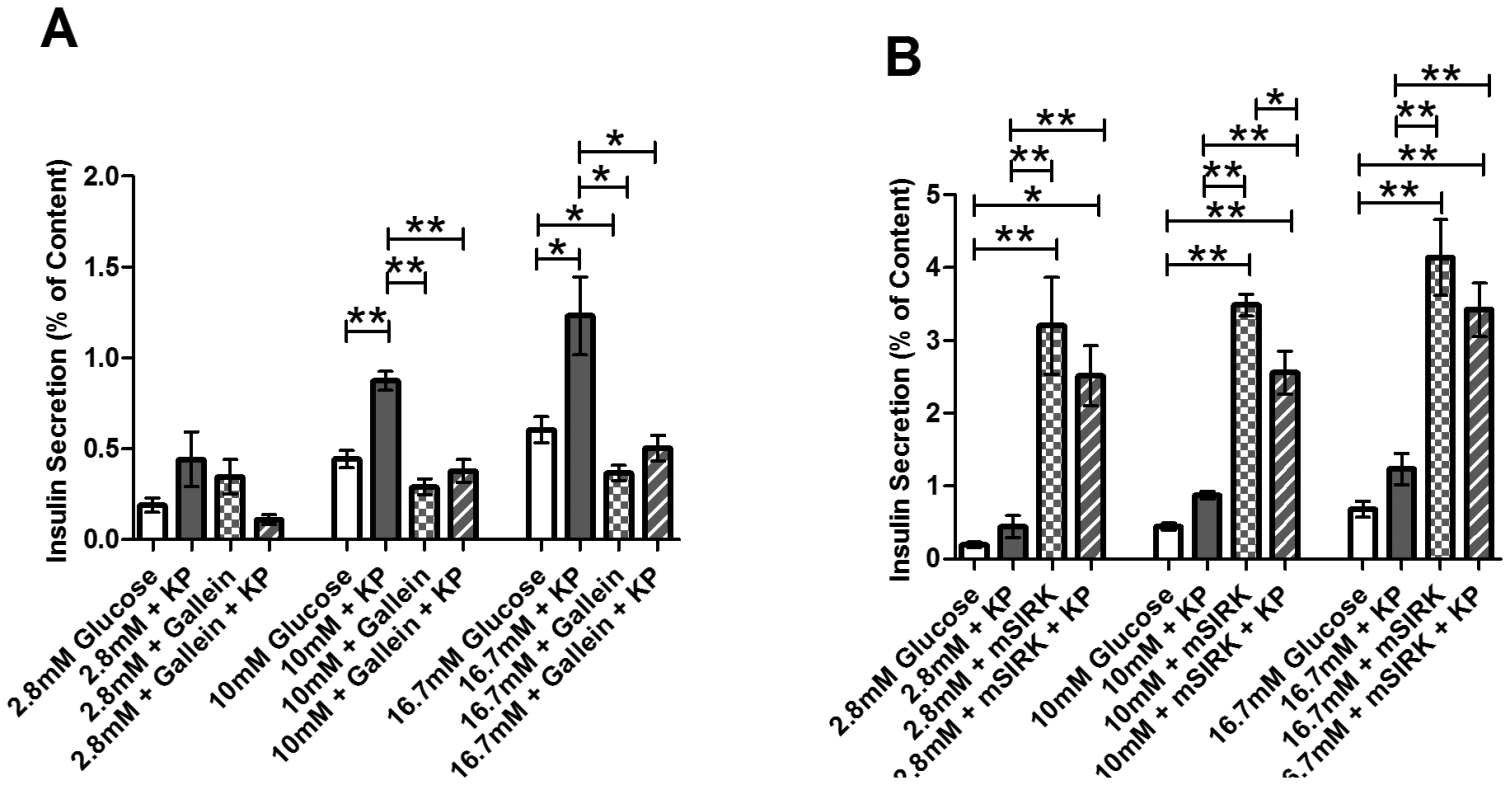
Percent of insulin content secreted from intact islets after incubation at 2.8, 10, or 16.7 mM glucose with and without treatment. Untreated control samples are shown in white. **A**, Percent of insulin content secreted at 2.8, 10, and 16.7 mM glucose concentrations in the presence and absence of KP (10 µM, dark gray), gallein (10 µM, checked), or gallein+KP (striped). **B**, Percent of insulin content secreted at 2.8, 10, and 16.7 mM glucose concentrations with and without KP (10 µM, dark gray), mSIRK (30 µM, checked), or mSIRK+KP (striped). Data are the mean ± S.E. *n* = 4–19. *p<0.05 and **p<0.001 compared to untreated control, KP alone, or mSIRK only.

Next, we examined whether KP’s stimulatory effect on insulin secretion is mediated through the G_α_ subunit or the G_βγ_ complex. Islets were treated with KP alone or combined with gallein or mSIRK at 2.8, 10, and 16.7 mM glucose levels. As previously shown [17], gallein alone inhibits insulin secretion at 16.7 mM glucose (*p*<0.02, Fig. 5A), and mSIRK increases insulin release at 2.8, 10, and 16.7 mM glucose compared to the untreated control islets (*p*<0.005, Fig. 5B). Incubation with mSIRK (L9A), the inactive analog of mSIRK, has been observed to yield secretion amounts at 2.8 mM and 16.7 mM glucose that are comparable to control [17]. At 2.8 mM glucose, gallein and KP combination treatment is not significantly altered compared to untreated control (*p*>0.1, Fig. 5A). Gallein and KP co-incubation significantly decreases insulin release at 10 mM and 16.7 mM glucose levels compared to kisspeptin alone (0.38±0.06% vs. 0.87±0.05% and 0.50±0.07% vs. 1.23±0.21%, *p*<0.001). These values are similar to those observed with gallein treatment alone (Fig. 5A). When islets were co-treated with mSIRK and KP, we noted no significant change compared to mSIRK alone at either 2.8 or 16.7 mM glucose concentrations (*p*>0.3, Fig. 5B); however, there is a slight reduction in insulin secretion upon combination treatment with mSIRK and KP at 10 mM glucose (*p*<0.05, Fig. 5B).

### GLP-1 signals through a G_α_ subunit

**Figure 6:**
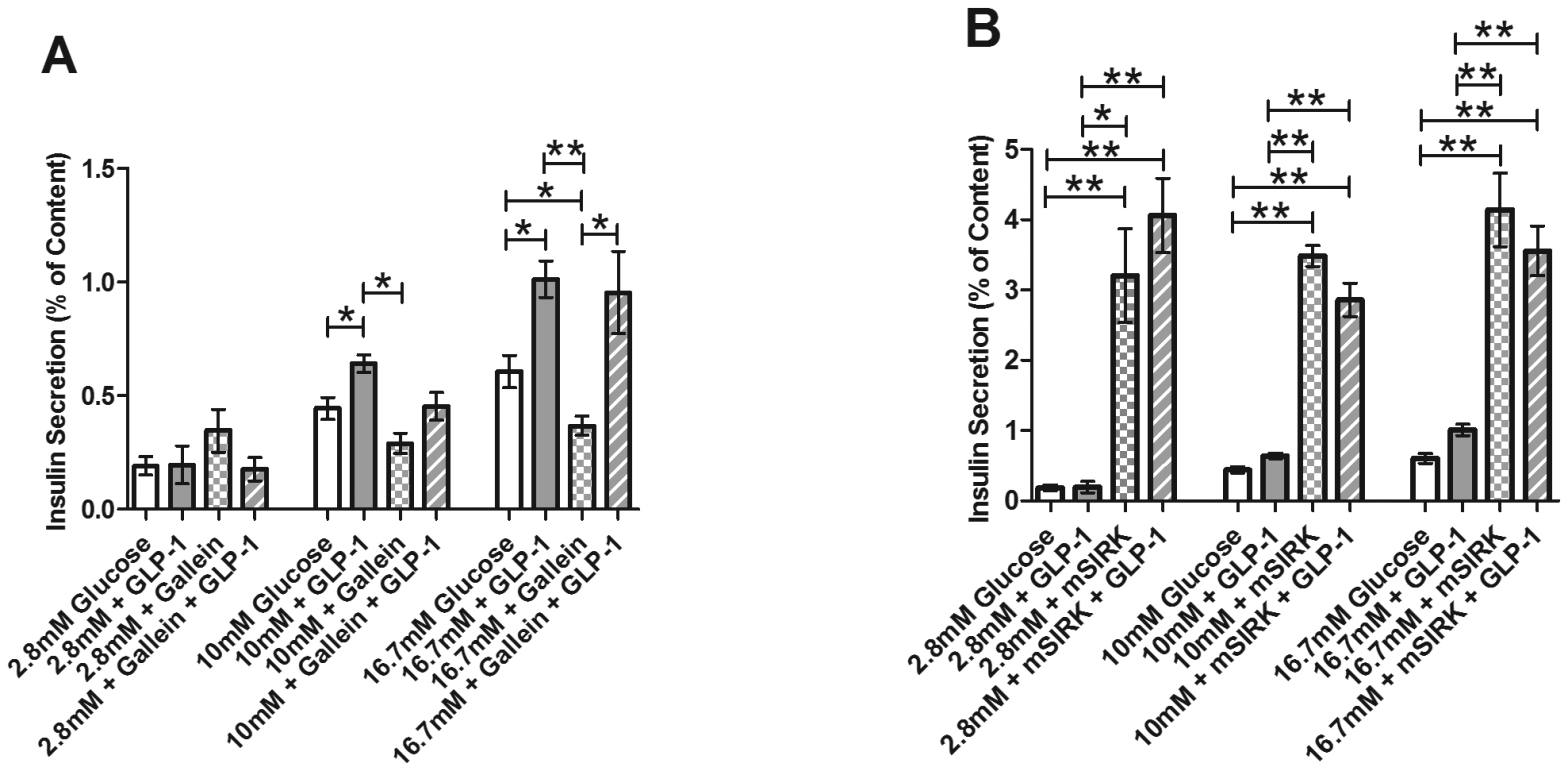
Percent of insulin content secreted from intact islets after static incubation at 2.8, 10, and 16.7 mM glucose with and without treatment. Untreated control samples are shown in white. **A**, Percent of insulin content secreted at 2.8, 10, and 16.7 mM glucose concentrations in the presence and absence of GLP-1 (20 nM, light gray), the G_βγ_ inhibitor, gallein (10 µM, checked), or combination treatment with gallein and GLP-1 (striped). **B,** Percent of insulin content secreted at 2.8, 10, and 16.7 mM glucose concentrations with GLP-1 (20 nM, light gray), the G_βγ_-activating peptide mSIRK (30 µM, checked), or combination treatment with mSIRK and GLP-1 (striped). Data are the mean ± S.E. *n* = 4–19. *(*p*<0.05) and **(*p*<0.001) indicate significance compared to untreated control, GLP-1 only, or gallein alone.

To determine whether GLP-1’s stimulatory effect on insulin secretion is also mediated through the G_βγ_ subunit, we incubated islets at 2.8, 10, and 16.7 mM glucose in the presence and absence of GLP-1 and gallein or mSIRK. At 2.8 mM and 10 mM glucose concentrations, GLP-1 in combination with gallein does not affect insulin secretion. Gallein and GLP-1 co-treatment at very high (16.7 mM) glucose did not alter secretion levels compared to GLP-1 alone (*p*>0.7), but they were significantly increased compared to gallein alone (*p*<0.005, Fig. 6A); there was also a trend toward an increase compared to the untreated control. When islets were treated with mSIRK and GLP-1, the data were not significantly altered compared to mSIRK alone at all glucose concentrations tested (*p*>0.1, Fig. 6B).

## Discussion

### Kisspeptin: G_βγ_-dependent increase in insulin release

Previous studies suggest that KP increases insulin secretion by activation of GPR54 receptors, which stimulates the PLC pathway. However, the mechanism by which KP modulates insulin secretion is poorly understood and there still is some debate as to the exact impact of KP on insulin release – whether it stimulates [6], [7] or inhibits [8], [9], [19] the response. While several reports showed that kisspeptin, especially at low concentrations, inhibits insulin secretion from perfused rat pancreas and intact mouse islets, respectively [8], [9], [19], we measured an increase in secretion (Fig. 1A), consistent with other previous reports from mouse and human islets [6], [7]. One confounding factor may include the use of kisspeptin 13 and/or kisspeptin 54, which are not endogenously expressed in mice; kisspeptin 10 (KP) and kisspeptin 52 are expressed in mice [32]. Our use of the endogenous peptide (kisspeptin 10, KP) at higher concentrations (1 µM) may, in part, account for the differences observed between our work and some of the previous reports. Altogether, these data suggest that not only the specific KP variant, but also the concentration of KP used, contribute to the stimulatory or inhibitory properties of the hormone.

Insulin secretion is tightly coupled to glucose metabolism. During metabolism of glucose, β-cells produce the reduced pyridine nucleotides NADH and NADPH (NAD(P)H) through glycolysis and the TCA cycle. The autofluorescence of these byproducts, which serves as an index of the cellular redox state and, thus, cellular metabolism, can be quantitatively measured using two-photon excitation microscopy [18], [22]. Here, we determined that KP increases the β-cell redox potential, suggesting that KP may potentiate insulin secretion, at least in part, through an enhancement of metabolic signaling (Fig. 1B). This data is consistent with the central dogma of insulin secretion stating that an increase in glucose metabolism will shift the ATP/ADP ratio to favor the production of ATP, which inhibits the ATP-sensitive inward rectifying potassium (K_ATP_) channel. This depolarizes the cell and allows for activation of voltage-gated Ca^2+^ channels (VGCC) [1]. Further, it has previously been shown that neither the G_βγ_ inhibitor, gallein, nor the G_βγ_-activator, mSIRK, significantly effect NAD(P)H levels compared to untreated control [17], suggesting that G_βγ_ activation can modulate insulin release through a pathway independent of glucose metabolism. The slight, but not statistically significant, increase in insulin secretion with KP treatment at low glucose (2.8 mM; Fig. 1B) supports the independence of KP’s actions from glucose stimulation. Thus, it is also possible that KP increases insulin secretion through a metabolism-independent mechanism, perhaps by altering diacylglycerol (DAG) effects on the secretory machinery (*i.e.* Munc proteins) or by activating protein kinase C (PKC) through direct G_βγ_ action; additional studies should be performed to further test this.

In β-cells, insulin secretion is regulated by changes in [Ca^2+^]_i_ [33], [34], so increased [Ca^2+^]_i_ elicits insulin release. Thus, we measured the Ca^2+^ response upon application of KP in both whole islets and dispersed β-cells, but detected no significant effect (Fig. 2 & 3). Bowe, *et al.* performed similar experiments in dispersed β-cells and found KP increases [Ca^2+^]_i_ [7]. There are several possible reasons for the differences between our data and that previously described. Dispersed β-cells respond differently to glucose stimulation than those in intact islets [35]–[37]. The Ca^2+^ response may be modulated by the loss of juxtacrine or paracrine signaling upon dispersion. Also, proteolytic damage to the cell during dispersion may modify the expression of cell surface proteins responsible for detecting changes in the extracellular environment. The differences observed between the Bowe, *et al.* study and our findings with dispersed β-cells may be due to protocol differences. Because we do not observe a significant effect of KP on [Ca^2+^]_i_ oscillation frequency or the amplitude of the oscillating component, the data suggest that KP does not exert its stimulatory effect on β-cell exocytosis by extensively modulating ER Ca^2+^ release. Upon co-application of KP and gallein, an inhibitor of G_βγ_ activity, or mSIRK, a G_βγ_-activating peptide, we also do not observe an effect on the Ca^2+^ response. Previously, our lab showed that mSIRK treatment increases the frequency of [Ca^2+^]_i_ oscillations [17]. Therefore, KP corrects the mSIRK-induced increase in Ca^2+^ activity. Because no change was detected with KP alone, the data suggest that the KP signaling pathway is independent of Ca^2+^.

Our insulin secretion data suggest that KP potentiates insulin release through a process that functions primarily through the G_βγ_ complex. As shown in Figure 6, KP co-treatment with gallein significantly decreases insulin secretion at stimulatory (10 mM and 16.7 mM) glucose concentrations. Thus, inhibition of G_βγ_ signaling eliminates the KP-induced stimulation of insulin release. No change is detected between mSIRK alone and mSIRK and KP in combination at 2.8 mM or 16.7 mM glucose. While a slight decrease in insulin secretion is detected with mSIRK and KP co-treatment compared to mSIRK alone at 10 mM glucose, secretion is still greatly potentiated compared to KP alone or the untreated control. Thus, mSIRK may maximally or near maximally stimulate insulin release. Together, the data support a model that the stimulatory effect of KP on insulin secretion is likely through G_βγ_-dependent activation of a Ca^2+^-independent process that stimulates glucose metabolism.

### GLP-1 potentiates insulin release through a metabolism and Ca^2+^ independent G_α_ pathway

GLP-1 potentiates GSIS through activation of a G_s_-coupled receptor, putatively by stimulating adenylate cyclase (AC) and increasing cAMP. Here, we confirmed that GLP-1 stimulates insulin release from isolated murine islets (Fig. 1A), and similar to previously published data, we noted that GLP-1 does not alter insulin secretion at sub-stimulatory glucose concentrations [11]. Unlike KP, β-cell redox potential is not significantly altered upon treatment with GLP-1 (Fig. 1B). This data supports a previous study which reported that GLP-1 and the GLP-1 mimetic Exendin-4 stimulate GSIS through a mechanism that does not alter β-cell metabolism [20]. Together, these data support the conclusion that GLP-1 modulates insulin secretion at stimulatory glucose concentrations at least partially through a metabolism-independent process.

To further investigate the mechanism of GLP-1 action, we measured β-cell Ca^2+^ activity prior to and following application of GLP-1. The whole islet Ca^2+^ response to increased glucose was not altered upon addition of GLP-1 (Fig. 4A & B). This novel finding is in contrast to previous studies using INS-1 cells and dispersed β-cells, which have shown that [Ca^2+^]_i_ increases upon addition of GLP-1 [30], [38], [39]. This difference highlights the limitations of tissue culture models and dispersed β-cells for mechanistic signaling studies, since β-cells require their islet microenvironment for normal function [40]. Our work utilized β-cells in intact islets to minimize these possible complications, and better mimic the *in vivo* conditions. However, we also measured the Ca^2+^ response in dispersed β-cells and our results were similar to those found in previous studies – GLP-1 treatment increased the Ca^2+^ activity in dispersed β-cells (Fig. 3) [32], [38], [39]. These data confirm the differences in Ca^2+^ activity observed between dispersed β-cells and those in intact islets. Further, co-treatment with GLP-1 and gallein or mSIRK, two G_βγ_ modulators, did not affect the frequency or the amplitude of the oscillating component. This result is similar to that observed with KP and suggests that GLP-1 treatment overcomes the mSIRK-induced increase in Ca^2+^ activity.

Insulin release upon incubation at 2.8, 10, and 16.7 mM glucose concentrations with GLP-1 and gallein was measured (Fig. 6A). Unlike with KP, no change is detected between GLP-1 and co-treatment with GLP-1 and gallein. Thus, inhibition of the G_βγ_ pathway does not alter GLP-1-induced insulin release. Combination treatment of mSIRK and GLP-1 also does not modulate insulin secretion compared to mSIRK alone (Fig. 6B). As similarly postulated with KP, mSIRK may maximally potentiate insulin secretion, thus explaining the absence of an additional effect with GLP-1 and mSIRK co-treatment. Collectively, our data suggest that GLP-1 increases insulin secretion from the β-cell at least partially through metabolism- and Ca^2+^-independent pathways; this process may be G_α_-dependent and occur further downstream than glucose metabolism and Ca^2+^ activity. However, it should be noted that further investigation is necessary to provide additional insight into the complete signaling pathway by which GLP-1 modulates insulin secretion.

We have shown the differential mechanisms by which KP and GLP-1 stimulate insulin secretion. Potentiation of cellular metabolism upon treatment with KP likely is mediated through a G_βγ_-dependent mechanism, perhaps linked to PLC or direct PKC activation, but independent of [Ca^2+^]_i_. GLP-1 activation of GLP-1R appears to act through the G_α_ subunit to stimulate a metabolism- and Ca^2+^-independent pathway. The data presented here reinforce the necessity of using whole islets to examine β-cell function, as the islet microenvironment and cell-cell communication have a tremendous impact on the secretion signaling pathway [4]. Given the abundance of available therapies targeting GPCRs, determining the mechanism(s) by which KP and GLP-1 signal in the islet may allow for a more comprehensive understanding of the possible therapeutic uses of this class of ligands.
